# Preparation and Digestive Properties of Biscuits Enriched with Extrusion-Modified Dietary Fiber: Effects on Slow Transit Constipation

**DOI:** 10.3390/foods14193436

**Published:** 2025-10-08

**Authors:** Zhan Wang, Dong Tan, Kemeng Zhao, Wangyang Shen, Jie Zhu, Hongjian Zhang, Xiwu Jia

**Affiliations:** 1 Department of Food Science and Engineering, Wuhan Polytechnic University, Wuhan 430023, China; wz@whpu.edu.cn (Z.W.);; 2 Key Laboratory for Deep Processing of Major Grain and Oil, Ministry of Education, Wuhan 430023, China; 3 COFCO Haijia (Xiamen) Noodle Industry Co., Ltd., Xiamen 361001, China; 4 Hainan Institute of Grain and Oil Science, Qionghai 571400, China

**Keywords:** wheat bran dietary fiber, biscuits, physical properties, digestive health

## Abstract

Dietary fiber (DF) is essential for digestive health, and wheat bran is a potential source because of its high fiber content. Extrusion processing enhances wheat bran’s functional properties by modifying its structure. This study aimed to examine the effects of extrusion-modified wheat bran dietary fiber (E-WBDF) on biscuits, focusing on textural, color, and digestive characteristics, and evaluate its ability to alleviate constipation using a mouse model. E-WBDF-enriched biscuits exhibited lower brightness, deeper color, reduced hardness, and a significant decline in digestion rate compared with conventional biscuits. In the mouse model, E-WBDF biscuits increased fecal volume and moisture, shortened defecation time, and accelerated small intestine transit. The results indicate that E-WBDF can enhance the physical properties of biscuits while reducing their digestion rate, thereby exhibiting a potential therapeutic effect in alleviating constipation in the mouse model. This study provides novel insights into using E-WBDF in biscuit formulations, offering a promising strategy for developing functional foods that promote digestive health.

## 1. Introduction

Dietary fiber (DF) is an essential component of a well-balanced diet, as it supports digestive health and helps reduce the risk of chronic diseases [1]. Among DF sources, wheat bran (WB) is notable for its high insoluble fiber content, which is crucial in enhancing bowel regularity and promoting a healthy gut microbiome [2]. Despite its health benefits, incorporating WB into food products can pose several challenges, including issues related to texture, solubility, and overall sensory acceptability [3]. These challenges arise because traditional WB can negatively affect the shelf life, processability, texture, and sensory properties of food products, making them less appealing to consumers despite their nutritional advantages [4].

Recent advancements in food processing technology have introduced extrusion modification to improve the functionality of WB [5]. Extrusion modification involves the application of heat and pressure to alter the physiochemical properties of the fibers, thereby enhancing its solubility and functional characteristics [6,7]. This process improves the integration of fiber into food matrices, making it more effective for increasing DF content while maintaining desirable sensory properties.

Biscuits are common baked snack foods made from cereal, appreciated for their ready-to-eat, natural, economical, practical, satiating, nutritional, extended shelf life, and crisp-tender qualities, making them one of the most consumed snack foods globally [8]. Being rich in carbohydrates and fats, biscuits can supply the body’s energy needs. Their convenient portability and extended shelf life make them ideal candidates for the development of functional foods [9]. DF can promote intestinal peristalsis, shorten the retention time of food in the intestines, and absorb water directly from the fiber, which helps soften stools and facilitates bowel movements. Consequently, the development and production of nutritious and health-promoting biscuits high in DF hold significant market potential [10,11].

Building upon our preliminary findings on extrusion-modified wheat bran dietary fiber (E-WBDF) [12], and incorporating the recent advances in DF modification [13] and functional food development [14], this study compared the textural, color, and digestive characteristics of biscuits before and after the addition of extrusion-modified DF. The constipation-relieving potential of E-WBDF biscuits was evaluated by monitoring fecal bulk, moisture, defecation duration, and small-intestinal transit time. Overall, this study aimed to provide a theoretical foundation for improving biscuit formulations and enhancing their functional benefits.

## 2. Materials and Methods

### 2.1. Materials and Reagents

Low-gluten flour was sourced from Xinxiang Liangrun Whole Grain Food Co., Ltd. (Xinxiang, China). WB was provided by COFCO Flour (Wuhan) Co., Ltd. (Wuhan, China). Butter was obtained from the Fonterra Co-operative Group. (Auckland, New Zealand). The powdered sugar was sourced from Taikoo Sugar (China) Co., Ltd. (Guangzhou, China). Whole milk powder was purchased from Nestlé (China) Co., Ltd. (Beijing, China). Trypsin, α-amylase, glucoamylase, and loperamide hydrochloride were obtained from Shanghai Yuanye Biotechnology Co., Ltd. (Shanghai, China). Male Kunming mice (6–8 weeks old) were obtained from the Hubei Province Centers for Disease Control and Prevention (Wuhan, China). Sterile saline was purchased from Sichuan Kelun Pharmaceutical Co., Ltd. (Chengdu, China), and India ink was obtained from Beijing Regen Biotechnology Co., Ltd. (Beijing, China).

### 2.2. Biscuit Preparation

Biscuit samples were prepared by first sifting together 0.6 g of ammonium bicarbonate, 1 g of baking soda, and 3 g of baking powder. These sifted ingredients were then mixed with 5 g of whole milk powder, one egg, 10 g of water, 30 g of butter, 1 g of salt, 30 g of sugar, and 15 g of E-WBDF. The mixture was thoroughly blended into 100 g of low-gluten flour to create a consistent dough. The dough was allowed to rest at room temperature before being molded into sheets. The sheets were then baked at 200 °C (top heat) and 190 °C (bottom heat) for 10 min, then cooled to 25 °C and subsequently sealed in plastic bags until analysis.

In this study, E-WBDF was prepared following our previously reported method [12]. The composition of E-WBDF was as follows: moisture content (4.46% ± 0.29%), protein (3.39% ± 0.09%), starch (0.12% ± 0.04%), lipid (1.38% ± 0.26%), DF (83.47% ± 0.38%), and ash (6.36% ± 0.19%).

### 2.3. Texture Measurement

After baking the two biscuit samples according to the specified recipes and procedures, they were allowed to cool and then cut into 1.0 cm × 1.0 cm squares. Texture analysis was performed using a TA-XT2i texture analyzer (Stable Micro Systems, Surrey, UK) equipped with a 36 mm-diameter cylindrical aluminum probe (P/36R). Samples were centered on the stationary stage and compressed once to 20% strain at 1 mm/s, with approach and retraction speeds of 2 mm/s and 1 mm/s, respectively; the trigger force was set at 20 g. Hardness, elasticity, and chewiness values were extracted from the resulting force–time curves.

### 2.4. Color Measurement

The surface color of the biscuit samples was measured using a Chromameter (Model CR-400; Minolta Co., Tokyo, Japan), calibrated against the supplier’s white ceramic tile. L*, a*, and b* coordinates were recorded. Each sample was assessed at three randomly selected points on the biscuit surface, and the mean values were calculated. The total color difference (Δ*E*) relative to a control or reference sample was determined using the following formula:

(1) $$\Delta E=\sqrt{{\left(L_{1}^{*}-L_{2}^{*}\right)}^{2}+{\left(a_{1}^{*}-a_{2}^{*}\right)}^{2}+{\left(b_{1}^{*}-b_{2}^{*}\right)}^{2}}$$

Here, $L_{1}^{*}$, $a_{1}^{*}$, and $b_{1}^{*}$ represent the color coordinates of the reference sample, whereas $L_{2}^{*}$, $a_{2}^{*}$, and $b_{2}^{*}$ represent those of the biscuit sample.

### 2.5. In Vitro Digestion of Biscuits

The in vitro digestion model included both gastric and intestinal phases [15]. The two biscuit types were ground and sieved through an 80-mesh screen for subsequent use. For gastric digestion, 1 g of biscuit powder (M) was suspended in 10 mL of simulated gastric fluid containing 0.9 mmol/L H_3_PO_4_, 0.15 mol/L NaCl, 0.1 mol/L HCl, 3 mmol/L CaCl_2_, and 16 mmol/L KCl (pH 2.5). Pepsin (3.6 g per 100 mL mixture) was then added, and digestion was conducted in a magnetic water bath at 37 °C with shaking at 170 rpm for 1 h. For intestinal digestion, the pH of the gastric digesta was adjusted to 6.5 using 20 mL of preheated (37 °C) simulated intestinal fluid (80.36 mmol/L NaHCO_3_, 1.5 mmol/L NaH_2_PO_4_, 0.7 mmol/L Na_2_HPO_4_, 0.49 mmol/L MgCl_2_, 54.46 mmol/L NaCl, and 4.56 mmol/L KCl; pH 7.0). To this mixture, 6 mg of trypsin, 65.9 mg of α-amylase, and 40 μL of glucoamylase were sequentially added, and then incubated in a magnetic water bath at 37 °C with shaking at 170 rpm for 3 h.

Samples (0.5 mL) were collected at 0, 10, 20, 30, 60, 90, 120, 150, and 180 min during intestinal digestion. Digestion was terminated by adding 10 mL of 10% trichloroacetic acid to each sample, followed by centrifugation at 10,000 rpm for 10 min. The glucose content in the supernatant was determined spectrophotometrically by the 3,5-dinitrosalicylic acid (DNS) method. A standard curve equation (Y = 3.3429X + 0.0207) was used to determine the glucose content (m) at each sampling point. The starch hydrolysis rate was calculated as the percentage of hydrolyzed starch relative to the total starch content using the indicated formula. This method provides a comprehensive assessment of the in vitro digestibility of the biscuit samples and offers insights into their potential nutritional benefits and digestive behavior.

### 2.6. Experimental Animal Models and Treatments

#### 2.6.1. Modeling of Slow Transit Constipation

Male Kunming mice (6–8 weeks old) were acclimatized for 1 week under controlled environmental conditions (temperature: 25 °C; relative humidity: 40–60%; 12 h light/dark cycle). Housing and experimental procedures were conducted in accordance with standard laboratory animal care protocols. Constipation modeling was performed according to the method described by Wang, with minor modifications [12]. Loperamide hydrochloride is widely used to induce constipation in rodents, as it reduces intestinal motility by activating μ-opioid receptors in the intestine, thereby inhibiting smooth muscle contraction. This results in delayed gastrointestinal transit, increased water absorption, and hardened stools, effectively simulating the physiological characteristics of human constipation. The mice were divided into four groups: control (saline), model (1.4 mg/mL loperamide hydrochloride solution), normal biscuit (1.4 mg/mL loperamide hydrochloride solution + 0.5 mg/L normal biscuit suspension), and E-WBDF biscuit (1.4 mg/mL loperamide hydrochloride solution + 0.5 mg/L E-WBDF biscuit suspension). The blank control group received daily oral gavage of distilled water (0.2 mL/10 g body weight) for eight consecutive days. On day 8, loperamide hydrochloride was administered to induce constipation, followed by treatment with E-WBDF via oral gavage at a dose of 0.5 g/kg for seven consecutive days. After the final administration, all mice were fasted for 24 h with ad libitum access to water. The gavage volume was maintained at 0.2 mL/10 g body weight. Constipation-related parameters were then assessed, including fecal water content, fecal pellet count, time to first black-stool defecation, and gastrointestinal transit rate.

This study was conducted in accordance with international guidelines for the ethical use of animals in research. The experimental protocol was approved by the Institutional Animal Care and Use Committee of Hubei University of Chinese Medicine (Approval No. HUCMS36047477).

#### 2.6.2. Mouse Defecation Experiment

Defecation kinetics were evaluated after a 12 h fast. The blank group received saline by oral gavage, whereas the remaining groups were administered loperamide hydrochloride solution followed immediately by the respective test suspension. Thirty minutes later, all mice were administered 0.2 mL of India ink via gavage and then returned to standard housing with free access to food and water, each housed individually in separate cages. The latency to the first black stool was recorded to the nearest minute. All feces excreted during the following 6 h were collected, counted, weighed (wet mass, *W*_1_), and subsequently dried at 60 °C to constant weight for moisture determination(dry weight, *W*_2_).

The fecal water content (*R*) was calculated as follows:

(2) $$R=\frac{\left(W_{1}-W_{2}\right)}{W_{1}}\times 100\%$$

#### 2.6.3. Propulsion Rate Determination in the Intestines

After a 12 h fast, mice received 0.2 mL of India ink via oral gavage 30 min after treatment. Twenty minutes later, they were euthanized via cervical dislocation under isoflurane anesthesia. The abdomen was immediately opened, and the entire intestinal tract from the pylorus to the anus was rapidly excised. The excised segment was gently straightened under minimal tension, and its total length (pylorus → anus) was recorded as *L*_1_, while the length of the ink-stained portion was recorded as follows:

(3) $$D=\frac{L_{2}}{L_{1}}\times 100\%$$

Here, *D* denotes the propulsion rate of the intestine, *L*_1_ represents the total length of the mouse intestine (cm), and *L*_2_ signifies the length of ink traveling within the intestine (cm).

### 2.7. Statistical Analysis

All measurements were performed in triplicate, and results are expressed as mean ± SD (*n* = 3). One-way analysis of variance, followed by Duncan’s multiple range test, was conducted using IBM SPSS Statistics 26 (IBM Corp., Armonk, NY, USA). Statistical significance was set at *p* < 0.05. Figures were generated using Origin 2022 (OriginLab Corp., Northampton, MA, USA).

## 3. Results

### 3.1. Biscuit Texture Analysis

To evaluate the effect of E-WBDF on biscuit texture, a comprehensive texture profile analysis was conducted, focusing on hardness, elasticity, and chewiness. As shown in Table 1, the addition of E-WBDF increased hardness, elasticity, and chewiness by 1274, 0.13, and 1713 gf, respectively. These changes are attributed to the enlargement of internal pore size and enhanced moisture retention, which influence the structural properties of the biscuits [16]. DF, with its high water-holding capacity, sequesters a substantial portion of the available aqueous phase, thereby restricting gluten hydration and impeding the formation of an extensive viscoelastic network. Additionally, DF modifies gluten network formation, which reduces dough cohesiveness and subsequently alters the final texture of the biscuits [17]. Overall, these findings highlight the significant impact of E-WBDF on the textural characteristics of biscuits.

### 3.2. Biscuit Color Analysis

Color is a crucial indicator of food quality, typically measured using a colorimeter, which provides L*, a*, and b* values. The influence of E-WBDF addition on the color properties of biscuits is presented in Table 2. The addition of E-WBDF significantly affected the color parameters of the biscuits (*p* < 0.05). The L* value decreased by approximately 12.5%, indicating a notable reduction in the brightness. Meanwhile, a* and b* values increased by approximately 42% and 8%, respectively, demonstrating a shift toward redder and yellower hues. These changes are ascribed to the natural pigments present in WBDF, such as polyphenols and carotenoids, and the intensified Maillard reactions and caramelization during baking. Consequently, the incorporation of E-WBDF significantly alters the visual properties of the biscuits, which may affect consumer perception and acceptability. The overall color difference, represented by the Δ*E* value, indicates that E-WBDF addition not only altered specific color parameters but also amplified the overall perceived color change, likely due to the more pronounced contrast introduced by WB pigments. This finding is consistent with the results reported by Ayoub et al. [18].

### 3.3. Biscuit Digestibility Characteristic Analysis

**Figure 1:**
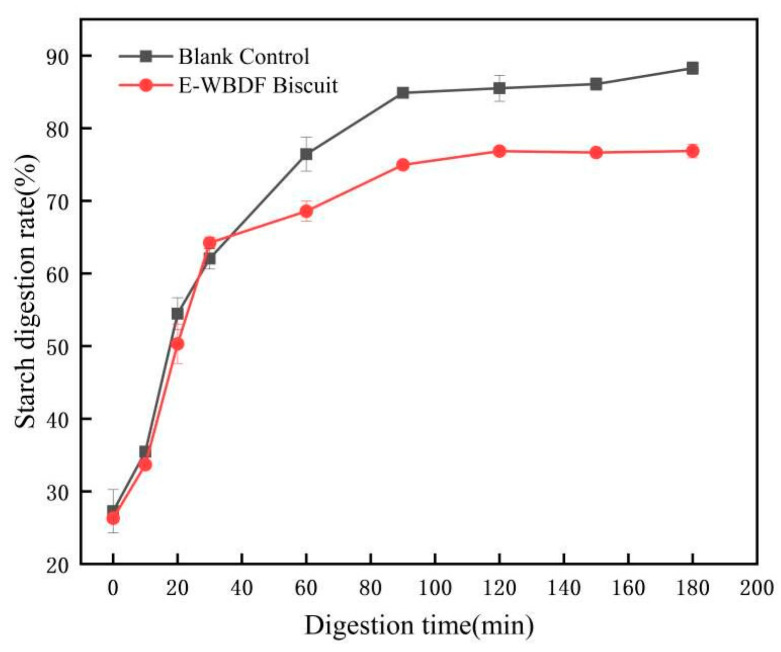
In vitro starch hydrolysis rate of biscuits. Blank control: Normal biscuit.

The in vitro starch hydrolysis rate of the biscuits is shown in Figure 1. The incorporation of the E-WBDF into the biscuits significantly reduced the starch hydrolysis rate, demonstrating its effectiveness in slowing digestion and decreasing starch breakdown. This reduction is attributed to the high fiber content, which inhibits starch digestion and glucose absorption. Over time, the enzymatic digestion rate stabilized, with a noticeable reduction in the hydrolysis rate after 90 min and a flatter digestion curve. This phenomenon can be explained by the formation of a protective fiber layer around the starch granules, which obstructs enzyme access and limits amylose release [19]. Consequently, biscuits containing modified WBDF exhibited a lower digestion rate (76.87%) than standard biscuits (88.27%). This finding indicates that high-fiber biscuits may support better postprandial blood glucose management and provide potential benefits for glycemic control and digestive health. These findings align with the results of previous studies highlighting the role of DF in modulating carbohydrate digestion and absorption [20,21]. To further elucidate the underlying mechanisms, additional research could explore the specific interactions between the fiber structure and enzymatic activity.

### 3.4. Animal Experiments

#### 3.4.1. Effects of Normal Biscuit and E-WBDF Biscuit on Defecation in Mice

Table 3 presents the time to first evacuation of black feces, number of fecal pellets excreted within 6 h, total fecal output within 6 h, and fecal water content in loperamide-induced constipated mice after treatment with different test samples. Compared with the control group, the model group exhibited clear signs of constipation: the first black stool was delayed by 66.25 min, 14.33 fewer pellets were excreted within 6 h, total fecal weight decreased by 0.23 g, and fecal water content decreased by 11.03%. These results confirm that the mouse constipation model was successfully established.

**Figure 2:**
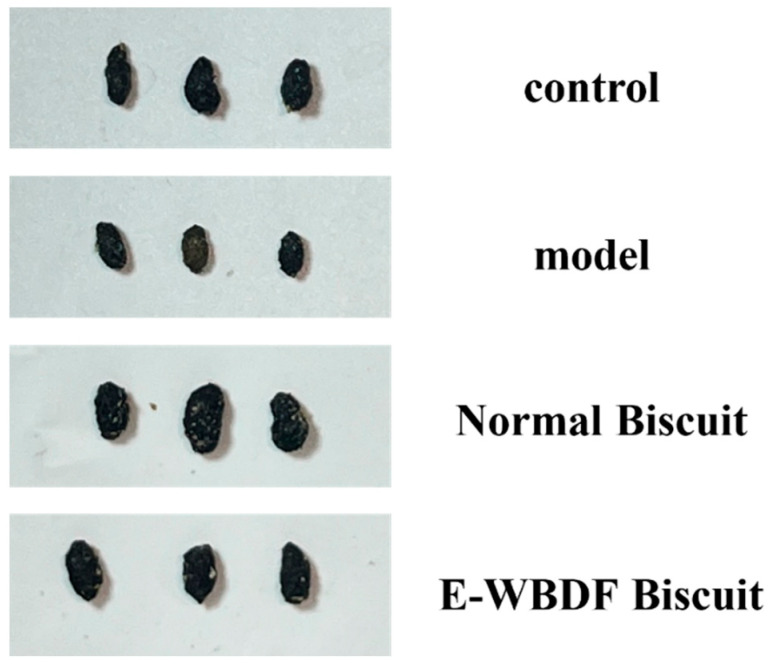
Fecal morphology of mice under different treatments.

The normal biscuit and E-WBDF biscuit groups demonstrated significant improvements in constipation symptoms by reducing the time to evacuate the first black feces and increasing the number of fecal pellets, total fecal output, and fecal water content (*p* < 0.05). The time to first evacuation of black feces in the E-WBDF biscuit group was 146.80 min, which was significantly shorter than that in the normal biscuit group (178.33 min, *p* < 0.05). Additionally, compared with the normal biscuit group, non-significant trends toward increased fecal pellet number, total fecal output, and fecal water content were also observed. Figure 2 shows the fecal morphology of mice under different treatments. The model group produced noticeably smaller feces than the control group, further confirming the successful establishment of the mouse constipation model. Conversely, the E-WBDF biscuit group produced markedly larger and moister feces (Figure 2), suggesting that E-WBDF increased fecal water content, stimulated intestinal motility, and facilitated defecation. The WB biscuit group showed more pronounced improvements, likely due to its higher DF content [22]. Overall, these results suggest that biscuits containing E-WBDF may alleviate constipation primarily by accelerating intestinal transit, with additional tendencies toward improved stool output and hydration.

#### 3.4.2. Effect of Normal Biscuit and E-WBDF on the Propulsion Rate of the Small Intestines in Mice

**Figure 3:**
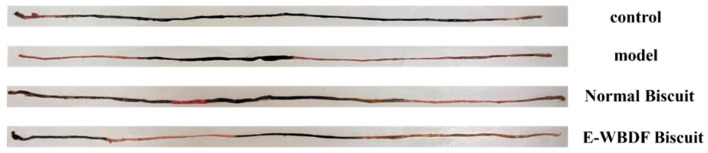
Small-intestinal morphology of mice under different treatments.

Table 4 and Figure 3 show the effects of different treatments on the total length of the small intestine, ink advancement distance, intestinal propulsion rate, and small-intestinal morphology. The control group exhibited the highest ink propulsion rate at 80.37%. Conversely, the model group showed a significantly reduced ink propulsion rate at 45.05%, indicating that the intragastric administration of loperamide hydrochloride weakened intestinal motility, successfully establishing a constipation model in mice. The normal biscuit and E-WBDF biscuit groups had ink propulsion rates of 58.22% and 66.11%, respectively, demonstrating an improvement over the model control group. This improvement can be attributed to the DF content of the biscuits. DF enhances intestinal motility by absorbing water and increasing the bulk of the intestinal contents, which stimulates peristalsis [23,24]. The WB biscuit group showed a 7.89% higher ink propulsion rate than the normal biscuit group. This is likely due to the higher DF content of WB biscuits, which can more effectively promote small intestine motility and prolong the gastric emptying time [25]. The increased propulsion rate in the E-WBDF biscuit group underscores the potential of DF in alleviating constipation and enhancing intestinal health, highlighting the importance of incorporating DF-rich foods to promote bowel regularity and prevent constipation.

## 4. Discussion

Compared with normal biscuits, E-WBDF-enriched biscuits exhibited reduced brightness, increased color depth, greater hardness, and a significantly lower starch digestion rate (reduced by 76%). These changes may be attributed to the incorporation of treated bran DF, which can promote Maillard reactions during baking and interfere with starch–gluten interactions, resulting in darker color, firmer texture, and restricted starch hydrolysis. Mouse experiments further demonstrated that compared with the normal biscuit group, the E-WBDF group passed the first black stool in a shorter time, excreted fewer fecal pellets within 6 h, and exhibited increased total fecal output, higher fecal water content, and an elevated ink propulsion rate. Collectively, these results indicate that E-WBDF is more effective than normal biscuits in alleviating constipation, as it exerts stronger laxative effects. Similar findings have been reported in previous studies showing that WB or soluble DFs enhance fecal bulk, increase water retention, and accelerate intestinal transit. Thus, the present study provides additional evidence that modified WB fiber can improve both the technological properties of biscuits and their physiological functionality.

## 5. Conclusions

In conclusion, incorporating E-WBDF into biscuits results in products exhibiting excellent overall performance and potential as dietary supplements for improving intestinal health. These findings establish both a theoretical framework and an experimental basis for the development of functional foods targeting chronic and episodic constipation, thereby advancing digestive-health-promoting product innovation.

## Figures and Tables

**Table 1:** Biscuit texture characteristics.

Sample	Hardness/gf	Elasticity/gf	Chewiness/gf
Normal biscuit	4507.19 ± 318.33 ^a^	0.49 ± 0.03 ^a^	2365.22 ± 263.07 ^a^
E-WBDF biscuit	5781.17 ± 227.87 ^b^	0.62 ± 0.04 ^b^	4078.43 ± 362.72 ^b^

Different letters in the same column (e.g., “a” and “b”) indicate significant differences (*p* < 0.05).

**Table 2:** Determination of the color difference in biscuits.

Sample	L*	a*	b*	Δ*E*
Normal biscuit	76.83 ± 2.41 ^a^	4.84 ± 1.78 ^b^	27.14 ± 1.72 ^a^	-
E-WBDF biscuit	67.21 ± 3.19 ^b^	6.87 ± 1.89 ^a^	29.40 ± 1.35 ^a^	10.09 ± 2.45

Different letters in the same column (e.g., “a” and “b”) indicate significant differences (*p* < 0.05).

**Table 3:** Fecal parameters of mice under different treatments.

Group	Time to First Evacuation of Black Feces/min	Number of Feces in 6 h/	Quantification of Fecal Output in 6 h/g	Fecal Water Content/%
Control	146.25 ± 2.86 ^c^	23.26 ± 4.19 ^a^	0.38 ± 0.03 ^a^	38.09 ± 2.56 ^a^
Model	212.50 ± 11.06 ^a^	9.33 ± 3.62 ^c^	0.15 ± 0.02 ^c^	27.06 ± 2.33 ^c^
Normal biscuits	178.33 ± 9.10 ^b^	13.33 ± 3.36 ^b^	0.20 ± 0.07 ^b^	32.85 ± 2.87 ^b^
E-WBDF biscuits	146.80 ± 11.30 ^c^	12.80 ± 4.19 ^b^	0.23 ± 0.03 ^b^	33.41 ± 1.93 ^b^

Data are presented as means ± standard deviation (*n* = 5); a, b, and c values in the same column indicate significant differences (*p* < 0.05).

**Table 4:** Propulsion rate of the small intestine in mice under different treatments.

Group	Total Length of the Small Intestine/cm	Ink Advanced Distance	Propulsion Rate/%
Control	45.25 ± 3.97 ^b^	36.20 ± 1.81 ^a^	80.37 ± 8.54 ^a^
Model	44.33 ± 4.17 ^b^	19.90 ± 1.22 ^c^	45.05 ± 1.86 ^d^
Normal biscuits	42.50 ± 1.70 ^b^	22.5 ± 3.24 ^b^	58.22 ± 3.31 ^c^
E-WBDF biscuits	50.50± 6.01 ^a^	33.57 ± 5.61 ^a^	66.11 ± 2.99 ^b^

Data are presented as means ± standard deviation (*n* = 5); a, b, c, and d values in the same column indicate significant differences (*p* < 0.05).

## Data Availability

The original contributions presented in this study are included in the article. Further inquiries can be directed to the corresponding author.

## Abbreviations

The following abbreviations are used in this manuscript:

DF	Dietary fiber
E-WBDF	Extrusion-modified wheat bran dietary fiber
WB	Wheat bran
